# Role of glycosylation in hypoxia-driven cell migration and invasion

**DOI:** 10.1080/19336918.2018.1491234

**Published:** 2018-08-19

**Authors:** Cecilia Arriagada, Patricio Silva, Vicente A. Torres

**Affiliations:** a Institute for Research in Dental Sciences, Faculty of Dentistry, Universidad de Chile, Santiago, Chile; b School of Pedagogy in Physical Education, Sports and Recreation, Universidad Bernardo O’Higgins, Santiago, Chile; c Advanced Center for Chronic Diseases (ACCDiS), Universidad de Chile, Santiago, Chile; d Faculty of Health Sciences, Universidad Central de Chile, Santiago, Chile

**Keywords:** Hypoxia, glycosylation, cell migration, integrins

## Abstract

Hypoxia, a common condition of the tumor microenvironment, induces changes in the proteome of cancer cells, mainly via HIF-1, a transcription factor conformed by a constitutively expressed β-subunit and an oxygen-regulated α-subunit. In hypoxia, HIF-1α stabilizes, forms the heterodimeric complex with HIF-1β, and binds to Hypoxia Response Elements (HRE), activating gene expression to promote metabolic adaptation, cell invasion and metastasis. Furthermore, the focal adhesion kinase, FAK, is activated in hypoxia, promoting cell migration by mechanisms that remain unclear. In this context, integrins, which are glycoproteins required for cell migration, are possibly involved in hypoxia-induced FAK activation. Evidence suggests that cancer cells have an altered glycosylation metabolism, mostly by the expression of glycosyltransferases, however the relevance of glycosylation is poorly explored in the context of hypoxia. Here, we discuss the role of hypoxia in cancer, and its effects on protein glycosylation, with emphasis on integrins and cell migration.

## Introduction

Cancer is the second most common cause of death worldwide, with an estimated of 171,2 deaths per 100,000 people per year, based on the 2008–2012 period, which is projected to continue increasing [1] . For this reason, it becomes important to study the molecular mechanisms involved in the development of tumors, in order to find new therapeutic targets. Altered glycosylation in human cancer cells has been acknowledged decades ago, and recent studies have increased our understanding about the role of protein glycosylation in tumor progression [2]. It has been suggested that the wide variety of changes in the glycoproteome depends on the origin and malignity of tumor cells, which could be due to transcriptional regulation of glycosyltransferases (GTs), the responsible enzymes for glycosylation [3]. Although, most studies have been focused on understanding the mechanisms by which glycosylation is altered in cancer cells, the effects of conditions in the tumor microenvironment and particularly hypoxia in the glycoproteome, is poorly explored.

Hypoxia is a common condition of the tumor microenvironment, which is associated with poor patient prognosis, enhanced tumor cell migration, invasion, and metastasis [4–6]. In this review, we will discuss the current state of the art about the relevance of hypoxia on protein glycosylation, and its consequences on tumor cell migration.

## Hypoxia in cancer

Low oxygen concentration or hypoxia is a potent microenvironmental factor observed in solid tumors that promotes metastasis [7], which is also associated with resistance to radiation therapy [8] and chemotherapy [9] . Normal oxygen concentration ranges from 19,7% to 3,8%, depending on a particular tissue; however, in solid tumors, oxygen concentration values are close to 0,3% to 2,1% depending on the tumor type [10,11]. In fact, the general consensus defines normoxia as the oxygen concentration commonly used in cell cultures (19,95%), whereas hypoxia is considered as any oxygen concentrations below physiologic normoxia (generally 1% oxygen) [10,11].

Cell adaptation to hypoxia involves the expression of a group of genes, many of which are transcriptionally regulated by the hypoxia-inducible factor 1 (HIF-1), which can induce changes in the proteome of tumor cells [12]. HIF-1, is a heterodimeric transcription factor consisting of a constitutively expressed β-subunit and an oxygen-regulated α-subunit. In normoxia, HIF-1α is hydroxylated on P402 and P564, which allows its interaction with the von Hippel-Lindau (VHL) E3 ubiquitin ligase, followed by its rapid polyubiquitynation and proteasomal degradation [13]. Alternatively, in hypoxia, HIF-1α is no longer degraded, allowing to its stabilization, and in a complex with HIF-1β, it binds to Hypoxia Response Element sequences (HRE; 5′[AG]CGTG-3′) localized in the promoter of HIF-1 target genes, to initiate gene expression [13]. More than 60 putative direct HIF-1 target genes have been identified, which could have a function in the development of the tumor phenotype [14]. Several groups, including our laboratory, have established that tumor cell migration and invasion are stimulated by hypoxia [15–20], and it has been suggested that hypoxia activates these phenotypes in tumor cells via multiple mechanisms, for example, through the direct and indirect regulation of the epithelial to mesenchymal transition (EMT) transcription factors Snail, Slug, Twist, and Zeb1 [21], or by promoting the invasion of surrounding tissues via upregulation and secretion of proteolytic enzymes, such as matrix metalloproteinases (MMPs), cathepsins, lysyl oxidases, and prolyl-4-hydroxylses (P4H) [22,23]. Another interesting mechanism whereby hypoxia increases cell migration is via activation of the Focal Adhesion Kinase (FAK) [15–17,24]. Our laboratory recently showed that hypoxia increases the phosphorylating activation of FAK (phosphorylation on Y397) in epithelial tumor cells, leading to sustained migration, invasion, and ECM remodeling [17]. Although the mechanisms underlying FAK activation by hypoxia are poorly understood, one possibility is that FAK is activated downstream of integrin receptors, which is supported by evidence showing that hypoxia induces the expression of integrins in several tumor cell models [25–28]. Interestingly, integrins and other cell surface proteins involved in cell migration, and known to be upregulated in hypoxia, are highly glycosylated [29,30]. Nevertheless, the role of hypoxia on protein glycosylation remains poorly understood.

## Glycosylation in cancer

Glycosylation is one of the most complex post-translational modifications, because it varies according to the expression of glycosylating enzymes, namely glycosyltransferases (GTs) [30,31]. GTs expression and their glycosylating activity depend on the cellular environment and the physio-pathological states, generating structural diversity on each glycoprotein repertoire [31]. The complete set of glycoproteins encoded by the genome is known as the cell glycoproteome, which can be structurally different between cells [31]. Changes in glycosylation, especially sialylation and fucosylation of glycans, are hallmarks of cancer cells, linked to an increase in oncogenic potential and invasiveness [2]. Alterations in glycan expression may be due to under or over-expression of GTs (Table 1). For instance, N-acetylglucosaminyltransferase V (GnT-V), sialyltransferases ST3Gal-I, ST6Gal1 and ST6GalNAc, and fucosyltransferases FUT1, FUT2, FUT3, FUT4 and FUT8 are often overexpressed in tumor cells (Table 1) [2,32]. Upregulation of these GTs leads to the expression of common tumor-cell epitopes such as sialyl-Lewisx and sialyl-Lewisa (sLex/sLea), Thomsen-nouvelle antigen (Tn), and sialyl-Tn (sTn), commonly used as tumor markers [33]. Additionally, it has been shown that GTs subcellular distribution is important for glycosylation. For instance, overexpression of ST6GalNAc-I, an enzyme that participates in sTn biosynthesis, leads to its accumulation throughout all Golgi cisternae, disrupting glycosylation by prematurely adding sialic acid to form the STn antigen [34]. Also, pH-dependent mislocalization of ST3 in Golgi, was correlated with impaired α(2,3)-sialylation of N-glycans [35].

**Table 1.** Regulation of glycosyltransferases in cancer. Glycosyltransferases upregulated or downregulated on cancer.

	Enzyme	Regulation
Sialyltransferases	ST3GAL1 ST3GAL3, ST3GAL4, ST3GAL6, ST6GAL1, ST6GALNAC1, ST6GALNAC2 ST8SIA1	Up-regulated
	ST3GAL3, ST3GAL 4 ST3GAL6, ST6GALNAC	Down-regulated
Fucosyltransferases	FUT1, FUT2, FUT3, FUT4, FUT5, FUT6 FUT7 FUT8 FUT9, FUT10 FUT11	Up-regulated
	FUT3, FUT4, FUT9, FUT10	Down-regulated
Others	- Glucosaminyl (N-acetyl) transferase (GNT1) - N-Acetylgalactosaminyltransferase 14 (GALNT14) - β-1,4-galactosyltransferase 1–2-3–5 (B4GALT) - Mannosyl (β1,4-)-glycoprotein β-1,4-N-acetylglucosaminyltransferas (MGAT3) - Mannosyl (alpha-1,6-)-glycoprotein β −1,6-N-acetyl-glucosaminyltransferase (MGAT5)	Up-regulated
	- Mannosyl (β1,4-)-glycoprotein β-1,4-N-acetylglucosaminyltransferas (MGAT3)	Down-regulated

## Glycosylation as a marker of malignancy

Increased branching of glycans and GTs expression have been associated with an increased malignancy in different tumor cells. Specifically, in breast cancer cells, upregulation of sialyltransferases, fucosyltransferases and N-acetylglucosaminyltransferases is associated with increased malignancy [36–39] metastasis and poor patient prognosis [40–42]. In colon cancer, augmented expression of mucins (heavily glycosylated proteins) and sialylated Tn and Lex antigens in tissues, is associated with increased metastasis [43], whereas increased fucosylation is associated with increased cellular adhesion [44]. Similar observations have been made in other tumors cells, such as liver, ovarian and melanoma cancer, among others, highlighting the importance of glycosylation in tumor malignancy, however, it remains poorly understood if microenvironmental conditions, such as hypoxia, could affect differentially protein glycosylation of malignant tumors cells [2].

## Glycosylation in hypoxia

Growing evidence suggest that hypoxia affects the expression of GTs in tumor cells, including human colon cancer cell lines SW480, C1 and Colo20, where a 7-day exposure to hypoxia was shown to increase the transcription of genes encoding for FUT7, ST3Gal-I and UDP-galactose transporter-1 (UGT1) [45]. Similarly, hypoxia promotes the transcription of FUT7, FUT1, GCNT2 and GCNT3 in immortalized prostate RWPE1 cells [46], although importantly, it remains unclear if HIF-1α is directly involved in such responses. The only evidence indicating that HIF-1α regulates GT expression was obtained by siRNA-based approaches, where HIF1α downregulation was followed by increased levels of mRNA encoding for FUT1 and FUT2, which was correlated with increased binding of the lectin UEA-1 and the antibody anti-LeY to the surface of HIFα-depleted cells [47]. However, whether HIF1α can bind to the promoter of GTs, allowing the transcription of GTs, and the putative effects of hypoxia on GTs protein levels and subcellular localization, remain largely unexplored. Consequently, the effects on protein glycosylation remain poorly understood.

Alternatively, protein glycosylation depends on both, monosaccharide synthesis and transport from cytosol to the ER and Golgi being central [48].UDP-GlcNAc, an intermediate necessary for N and O-linked glycosylation, is synthesized in the hexosamine pathway, from monosaccharide precursors, such as glucose [49]. Consequently, alterations in this biosynthetic pathway or in previous transport of precursors affect the glycosylation pattern. In this respect, changes in levels of the different transporters and enzymes involved in their synthesis, have been reported during hypoxia [50]. Specifically, hypoxia has been shown to increase the expression of glucose transporters GLUT1 and GLUT3, as well as the enzyme hexokinase-2 (HKII) in a variety of cell lines [50]. Moreover, hypoxia promotes the expression of the enzyme pyruvate dehydrogenase kinase 1 (PDK1) in human Burkitt’s lymphoma cell line P493-6, in a time-dependent manner [51]. Additionally, that study reported that HIF-1 directly regulates the expression of PDK1 by chromatin immunoprecipitation assays [51]. Alternatively, a Hypoxia Response Element (HRE) was found in the promoter of glycogen synthase 1 (GYS1), as shown in skeletal muscle myotube cells C2CL2, where HIF-1α binding allows the expression of GYS1, resulting in an increased activity and the accumulation of glycogen [52]. Other studies showed that HIF-1α controls glycogen levels by inducing the expression of PPP1R3C (also known as ‘protein targeting to glycogen’ (PTG)), via binding to a functional HRE sequence [53]. Also, mRNA levels of Glutamine-fructose-6-phosphate (GFPT1) transaminase increases during hypoxia, which correlates with an increased enzyme activity [54]. Intriguingly, despite Glycosylated Phosphofructokinase 1 (PFK1) levels are increased during hypoxia, PFK1 activity was shown to decrease [55].

Collectively, all these data suggest that hypoxia has a relevant role in protein glycosylation, either directly or indirectly, leading to altered functional activities. In this respect, it will be critical assessing the role of glycosylation-induced by hypoxia on proteins relevant to the migratory, invasive, and metastatic characteristics in tumor cells. This is particularly relevant, because several proteins involved in tumor cell migration and invasion are heavily glycosylated, those including integrins, metalloproteinases, and cadherins, amongst others [56,57]. Most importantly, several proteins from this group, specially integrins, are known to be deregulated in hypoxia [23,25]. Hence, the putative role of hypoxia on integrin glycosylation, is just beginning to be explored, and thus initial studies in the field will be discussed in the upcoming paragraphs.

## Integrin activation and trafficking

**Figure 1.**
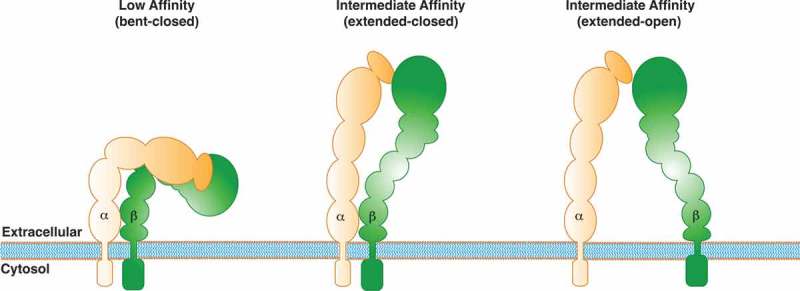
Schematic model of β_1_-integrin activation. The model shows different conformations of integrins, along with their different affinities: the bent conformation (low-affinity), and the two extended head-piece conformations (intermediate-affinity and high-affinity).

Integrins are a family of large transmembrane cell surface receptors composed of 18 α and 8 β subunits, which can form 24 different heterodimers and interact with different extracellular matrix ligands such as laminin, fibronectin and collagen, among others. Integrins regulate diverse cellular processes including cell survival, motility, and proliferation, and their contribution to cell migration and invasion is one of their most studied functions in tumor cell biology [58,59]. The function of integrins is controlled by activation and inactivation mechanisms of the receptor complex. In their inactive state, integrins are in a low-affinity closed conformation (bent) (Figure 1) [60]. Upon extracellular ligand binding (*outside-in signaling*), integrins undergo conformational changes, first to an intermediate affinity conformation (closed), and then to a high-affinity open conformation (extended), allowing to separation of the tail domain and subsequent interaction with cytoplasmic regulators (Figure 1) [60]. Alternatively, integrin signaling can be triggered by intracellular activators (*inside-out signaling*), such as talin or kindlin, which bind to the β tail, leading to conformational changes that increase the affinity to extracellular ligands [60]. After these initial events, integrin-containing adhesion complexes (referred to as focal adhesions, FAs) grow in size, and mature to form macromolecular complexes, which contain more than hundreds of different adhesion proteins, including FAK [60,61].

As for other cell surface receptors, integrin signaling is also regulated by the delivery of newly synthesized proteins to the cell surface, as well as their endocytosis from the plasma membrane, and their recycling or degradative trafficking routes [61–63]. It has been shown that inactive and active (ligand-bound) conformations of integrin heterodimers can be internalized via macropinocytosis [64], clathrin-dependent endocytosis [65] and clathrin-independent endocytosis, which includes caveolae-mediated endocytosis [66] and clathrin-independent carriers (CLICs) [67]. Intriguingly, regardless the endocytic mechanism, a subset of regulators of integrin trafficking has been extensively studied, namely a group of Rab small GTPases [61]. For instance, active and inactive β_1_-integrin are endocytosed through the clathrin and dynamin routes, allowing its co-localization with Rab5 and Rab21 in early endosomes, as shown in PC-3, MDA-MB-231 and NCI- H460 cells [68]. Also, in endothelial cells, the Ras and Rab Interactor 2 (RIN2) localizes to adhesion complexes and plays a role in the endocytosis of active β_1_-integrin [69]. On the other hand, endocytosed integrins traffic *en route* to late endosomes/lysosomes, or recycle back to the plasma membrane via recycling endosomes or alternative routes [61]. In this regard, active β_1_-integrin was shown to recycle via Rab11-dependent, long recycling loop, whereas the inactive conformation of this integrin is rapidly recycled in an Rab4-dependent manner [70]. Importantly, integrins have a long half-life (18 hours) when compared to the rates of their own recycling (30 minutes approximately, depending on the pathway), and several reports have demonstrated that non-recycled integrins are targeted for lysosomal degradation instead [61,70]. However, lysosomal targeting does not necessarily result in integrin degradation, because integrins can even recycle back to the plasma membrane from these compartments [68,70].

Alternatively, glycosylation has been proposed as a relevant post-translational mechanism contributing to integrin regulation [27–29]. Despite altered glycosylation has been proposed as a hallmark of cancer cells, and integrins are recognized as highly glycosylated proteins, only a few studies have addressed the effects of glycosylation on integrin expression, trafficking, localization and biological function. In this respect, current knowledge is rather limiting. The role of integrins in cancer cells is one of their most studied functions, because of their relevance in cell migration and invasion (reviewed in [71,72]). In this regard, a wide variety of integrins contribute to tumor progression and integrins known to be overexpressed in cancer include α6β4, α6β1, αvβ5, α2β1 and α3β1 [59]. Also, it has been suggested that several additional integrins are crucial for tumor angiogenesis, such as αVβ3, α1β1, α2β1, α4β1, α5β1, α6β1, α9β1 and α6β4 [59]. Noteworthy, several of these studies have pointed out to the relevance of β_1_-integrin in cancer (reviewed in [73]), and inhibition of this specific integrin with blocking antibodies, significantly impaired tumor growth *in vitro* and *in vivo*, which was also paralleled with antiangiogenic effects [74,75]. These compelling data highlight the central role of β_1_-integrin in tumor progression.

## Effects of glycosylation on integrin function

**Figure 2.**
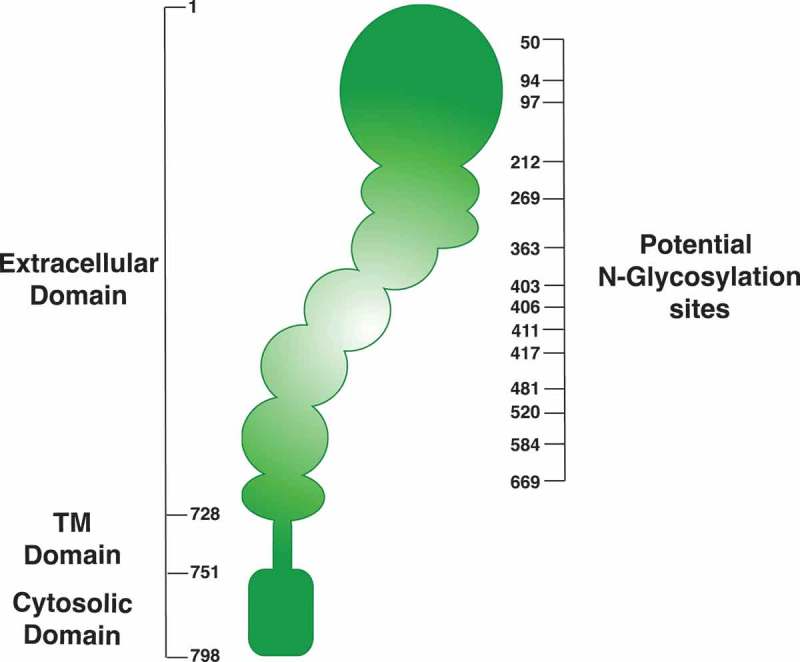
**Schematic diagram of potential N-glycosylation sites of β_1_-integrin**. Data was obtained using the Uniprot data base. http://www.uniprot.org/uniprot/P05556.

Protein glycosylation is central to different cellular functions, because it affects protein folding, trafficking, subcellular localization, and degradation [76]. In this regard, accumulating evidence suggest that integrins possess over 20 potential N-glycosylation sites (Figure 2 shows a schematic summary of potential glycosylation sites, obtained from the Uniprot data base), and that N-glycosylation is crucial for integrin function [27,29,30]. Specifically, β1,6 branching of N-linked oligosaccharides on β1-integrin is important for both function and structure, because it is required for β1 clustering and interaction with the ECM [77]. Also, hyposialylation (i.e. specific changes in glycosylation branching size) of β1-integrin increases its binding to fibronectin and cell adhesion [78]. Moreover, overexpression of GnT-III blocked α5β1-mediated cell spreading, migration and FAK phosphorylation [79], suggesting that modification of the N-glycan branch size affects integrin downstream signaling and function. In this work, the authors focused on the effects of the overexpression of GnT-III on α2, α3 and α5, which substantially increased its reactivity to lectin E4-PHA in transfected cells.

Finally, it has been shown that complete ablation of putative N-glycosylation sites in β1-integrin, by treatment with N-glycosidase F and tunicamycin, decreases integrin cell surface level and binding to fibronectin, respectively [80], whereas the ablation of glycosylation sites by mutagenesis leads to decreases in the cell surface levels of α5 [81] and β1 [82,83] and sustained FAK activity [81–83]. However, it remains unclear how specific changes in the glycosylation sites and their branching size could affect integrin subcellular localization, and most importantly, their regulation in the context of hypoxia, remains largely unexplored.

## Effects of hypoxia on integrins

Hypoxia has been shown to enhance the expression of integrins, including β_1_-integrin in fibroblasts and keratinocytes [26,84], α_v_β_4_ in breast carcinoma cells [28], β_3_ and β_5_ integrins in endothelial cells [85], α_v_β_3_ in melanoma cells [24], β_2_ in leukocytes [86] and the α_5_ subunit in colon cancer cells [45]. Also, in human mesenchymal stem cells, hypoxia increased RNA expression of various integrins (α_1_, α_3_, α_6_, α_11_, α_v_, β_1_, β_3_) [87]. However, the underlying mechanisms involved in such regulations by hypoxia remain to be assessed. Specifically, it has been shown that HIF-1 and HIF-2 bind to the promoter of α_5_-integrin under hypoxic conditions [88], and that HIF-1 binds to the promoter of β_1_-integrin [26], however, whether hypoxia alters post-translational events leading to integrin expression and function, such as their trafficking and degradation, is not clear. The only available study that connects integrin glycosylation with hypoxia, showed that N-glycosylation of α_3_-integrin subunit is upregulated in A431 squamous carcinoma cells during hypoxia, leading to increased integrin surface levels, and enhanced cell invasion [89]. In the same work, the authors found an increase in overall β_1_-integrin glycosylation in hypoxia, however, they did not determine if hypoxia-dependent glycosylation on β_1_-integrin affects its subcellular localization or function [89]. Most importantly, the nature of the glycosylation sites, patterns, and branching sizes, were not explored. Collectively these pieces of evidences suggest that integrin glycosylation is a key event accounting for hypoxia dependent cell migration; however, determining the molecular mechanisms and precise residues undergoing glycosylation is a demanding issue.

## Conclusions

**Figure 3.**
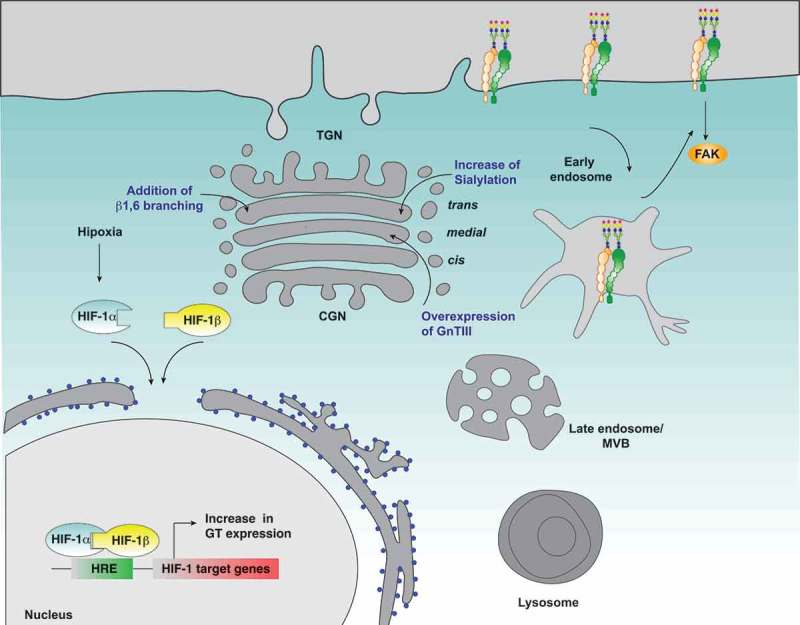
Possible effect of hypoxia on β_1_-integrin glycosylation and cell migration. Hypoxia induces the expression of glycosyltransferases in a HIF1α dependent manner, which could lead to changes in the glycosylation profile of β_1_-integrin, and thus affecting its subcellular distribution and function, its interaction with extracellular ligands, FAK signaling and cell migration. In blue letters are indicated the main modifications on β_1-_integrin glycosylation that affect its function and the Golgi compartments where they occur.

Hypoxia is one of the most important factors within the tumor microenvironment as it is a determining factor in the prognosis of patients. However, to date, the mechanisms by which hypoxia controls tumor progression are not yet fully understood. Nevertheless, growing evidence support the roles that hypoxia and HIFs play in the control of tumor cell migration, invasion and metastasis. Despite that several proteins controlled by hypoxia and HIFs have been determined, the role of glycosylation and the expression of GTs in the biology of cancer in the context of hypoxia have been poorly addressed. Here, we spotlight the recent literature that shows the importance of protein glycosylation under the context of hypoxia, specifically in key proteins associated to cell migration and invasion. In this context, our focus was on integrins, since this family of cellular surface proteins offers many possibilities for regulation in the context of glycosylation, such as their own trafficking and subcellular localization, as well as their stability, activity status and downstream signaling (Figure 3). Despite this interesting approach, limited studies have shown the regulation of glycosylation of integrins in hypoxia and its role in the induction of cellular processes such as tumor cell migration and invasion. Even so, this hypothesis unfolds an interesting window for future design of therapeutic strategies that address cellular glycosylation regulated by hypoxia within the tumor context, specifically metastasis, that seek to control the cancer spread and improve the prognosis of patients.
